# Co‐immobilized Phosphorylated Cofactors and Enzymes as Self‐Sufficient Heterogeneous Biocatalysts for Chemical Processes

**DOI:** 10.1002/anie.201609758

**Published:** 2016-12-21

**Authors:** Susana Velasco‐Lozano, Ana I. Benítez‐Mateos, Fernando López‐Gallego

**Affiliations:** ^1^ Heterogeneous biocatalysis groupCIC biomaGUNE, Edificio Empresarial “C”Paseo de Miramón 18220009DonostiaSpain; ^2^ IKERBASQUE, Basque Foundation for ScienceBilbaoSpain

**Keywords:** biocatalysis, cofactor regeneration, heterogenous catalysis, porous materials, protein immobilization

## Abstract

Enzyme cofactors play a major role in biocatalysis, as many enzymes require them to catalyze highly valuable reactions in organic synthesis. However, the cofactor recycling is often a hurdle to implement enzymes at the industrial level. The fabrication of heterogeneous biocatalysts co‐immobilizing phosphorylated cofactors (PLP, FAD^+^, and NAD^+^) and enzymes onto the same solid material is reported to perform chemical reactions without exogeneous addition of cofactors in aqueous media. In these self‐sufficient heterogeneous biocatalysts, the immobilized enzymes are catalytically active and the immobilized cofactors catalytically available and retained into the solid phase for several reaction cycles. Finally, we have applied a NAD^+^‐dependent heterogeneous biocatalyst to continuous flow asymmetric reduction of prochiral ketones, thus demonstrating the robustness of this approach for large scale biotransformations.

Industrial biocatalysis is transforming chemical manufacturing towards more sustainable and environmentally friendly processes.^1^ Some of the most interesting reactions in industrial biocatalysis are catalyzed by cofactor‐dependent enzymes such as NADH‐dependent reductases and oxidases, PLP‐dependent transaminases, and FAD^+^‐dependent oxygenases.^2^ Consequently, the regeneration and reutilization of these expensive cofactors is a major requirement for the implementation of enzymatic processes at the industrial scale.^3^ Hitherto, many systems based on enzymatic and chemical reactions regenerate the cofactors allowing their use in catalytic amounts, but still they must be exogenously added. Inspired by nature, NAD(P)H and enzymes have been co‐immobilized on solid materials allowing their solid‐phase recycling and reusability for several operational cycles.3a, 4 These heterogeneous systems, however, have shown several drawbacks; the enzymatic activity towards the immobilized cofactors is low,^5^ the turnover numbers of the immobilized cofactors are poor (≤1),^6^ the re‐usability of both enzymes and cofactors is limited^5^, ^7^ and the fabrication of these systems is hardly scalable.^8^ However, the use of strong and non‐porous anionic exchangers has shown that the cofactor re‐utilization works in organic media^9^ but fails in aqueous media owing to the lixiviation of both NAD(P)H and enzymes from the matrix.^10^ The reason behind such lixiviation is the formation of reversible ionic interactions between the negatively charged phosphorylated cofactors and the positive charges of the solid material, establishing an association/dissociation equilibrium which permits the release of cofactor molecules from the solid surface to the bulk.^11^ In contrast, if we ionically adsorb the cofactors on porous materials, such adsorption is dynamic and allows that some cofactors are associated to the solid surface enabling their reutilization in aqueous media, while others are dissociated (free) but confined into the porous space becoming available for the enzymes. Therefore, the cofactors are continuously shifting from the associated to the dissociated states generating an exchange between the enzyme active sites and the carrier surface without being released to the bulk. Herein, we harness such association/dissociation equilibrium within a porous environment to fabricate self‐sufficient heterogeneous biocatalysts capable of regenerating and retaining the phosphorylated cofactors in the solid‐phase for several operational batch‐cycles and in continuous processes in aqueous media.

We first optimized the ionic adsorption of NAD^+^ on two different anionic exchangers under different conditions^12^ (Supporting Information, Table S1). Under a low buffer concentration (10 mm) and offering 100 μmol${}_{\mathrm{NAD}{}^{+}}$ g^−1^ at pH 7.0, agarose microbeads activated with polyethyleneimine 25 kDa (Ag‐GPEI) loaded 18 μmol${}_{\mathrm{NAD}{}^{+}}$ g^−1^ and retained 22 % of the immobilized cofactor after 5 wash cycles, whereas agarose microbeads activated with triethyl amine (Ag‐TEA) loaded 11 μmol${}_{\mathrm{NAD}{}^{+}}$ g^−1^ and 100 % of the cofactor was lixiviated after the first wash (Supporting Information, Table S1, Figure S1). Similar results were found when PEI was irreversibly attached to other porous commercial carriers (Purolite) (Purolite‐GPEI) and agarose microbeads activated with divinyl‐sulfone (Ag‐DVSPEI)^13^ (Supporting Information, Figure S2). Encouraged by these results, we expanded this strategy to other phosphorylated cofactors, such as flavin adenine dinucleotide (FAD^+^) and pyridoxal phosphate (PLP) used by other industrially relevant enzymes.2a,2c,2d, 14 Figure 1 A shows that the absorption yield of PLP on Ag‐GPEI was 1.8 and 4.8 times higher than FAD^+^ and NAD^+^, respectively, and 99 % of immobilized PLP remained in the microbeads after 8 washes with low ionic strength buffer at pH 7, while 85 % and 80 % of the adsorbed FAD^+^ and NAD^+^ were lixiviated under the same conditions, respectively (Figure 1 B). Both cofactor adsorption and lixiviation rely on the association/dissociation equilibrium that governs the interactions between Ag‐GPEI and each cofactor. The apparent dissociation constant (Kappd ) for PLP equilibrium is 26 and 6 times lower than for NAD^+^ and FAD^+^ equilibrium, respectively (Supporting Information, Table S2), which supports the notion that the equilibrium of the cofactor–PEI interactions favors 99 % of PLP remaining in the solid phase while FAD^+^ and NAD^+^ are significantly lixiviated after 8 washes (Figure 1 B).

**Figure 1:**
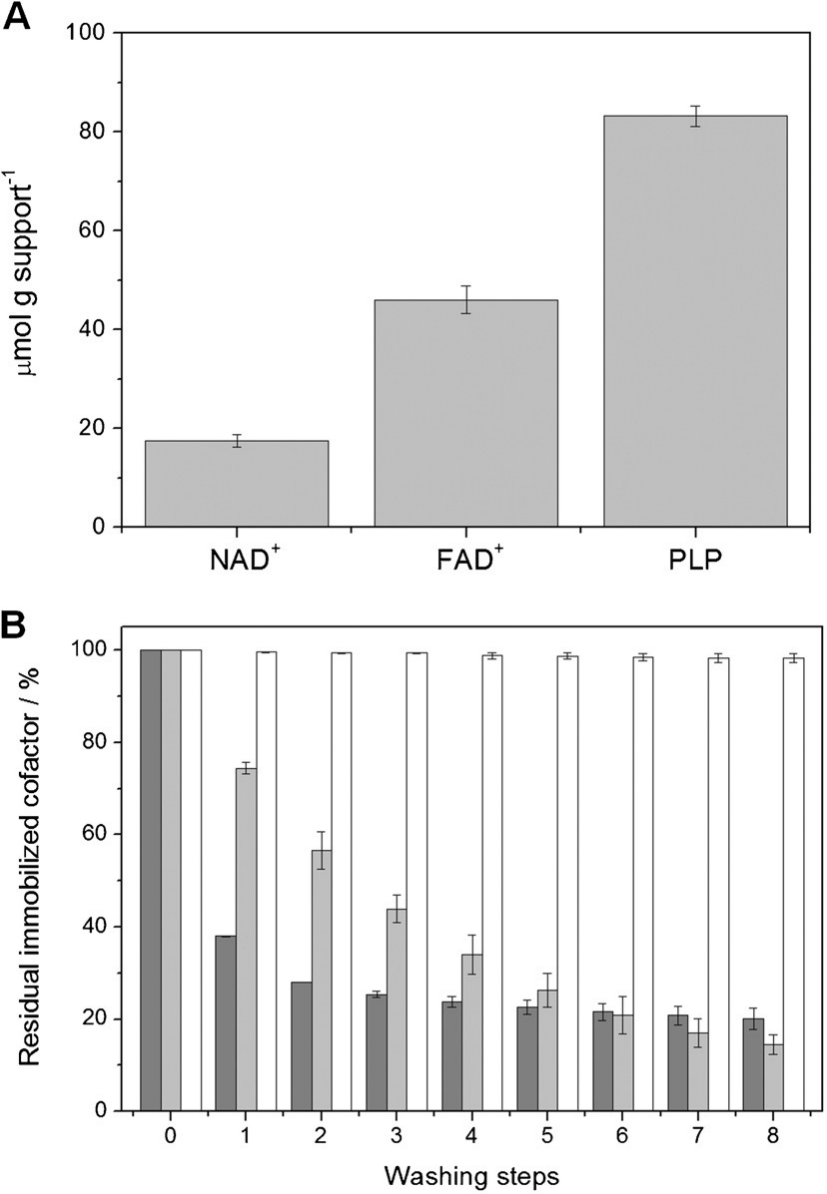
A)  Immobilization of phosphorylated cofactors on Ag‐GPEI (offered 100 μmol_cofactor_ g_support_^−1^). B) Residual NAD^+^ (dark gray bars), FAD^+^ (light gray bars), and PLP (white bars) bound to Ag‐GPEI after washing treatments with 10 mm sodium phosphate at pH 7.

Usually, enzymes display low activities towards reversible or irreversible tethered cofactors on solid materials.^5^, ^6^, 7b To test the performance of these reversibly immobilized cofactors, we studied the steady‐state kinetics of NAD^+^‐dependent formate dehydrogenase from *Candida boidinii* (Cb‐FDH) towards both soluble and immobilized cofactors (Supporting Information, Figure S3 A). Using soluble NAD^+^, the *K*_M_ of soluble Cb‐FDH was 12 times lower than its immobilized form (Supporting Information, Figure S4). Unfortunately, the apparent Michaelis–Menten constant could not be calculated using the immobilized NAD^+^ because neither the soluble nor the immobilized enzymes were saturated at any concentration of immobilized cofactor. Under limiting NAD^+^ conditions (166 μm in the bulk=6.6 μmol_cofactor_ g_support_^−1^) where enzyme activity linearly increases with the cofactor concentration, the soluble Cb‐FDH was 10 times less active towards the immobilized cofactor than towards the soluble one. In contrast, the immobilized enzyme was similarly active towards both soluble and co‐immobilized NAD^+^ under the same conditions (Supporting Information, Table S3). Introducing those activity values in the corresponding Michaelis–Menten equations, we estimated that apparent concentration of immobilized NAD^+^ that was catalytically available for Cb‐FDH was 30 times higher when the enzyme was co‐immobilized with the cofactor than when the enzyme was soluble (Supporting Information, Table S3). These results suggest that both the co‐immobilization of enzymes and cofactors and the association/dissociation equilibrium of NAD^+^ inside the porous microenvironment facilitate the cofactor diffusion from the polymeric layer to the Cb‐FDH active sites.

Having now in hand a versatile and simple strategy to incorporate phosphorylated cofactors within porous carriers, we co‐immobilized two different enzyme systems with their corresponding cofactors (Table 1); system **HB1** formed by NAD^+^, alcohol dehydrogenase 2 from *Thermus thermophilus* 15 (Tt‐ADH2; Supporting Information, Figure S3 B) and Cb‐FDH to carry out the asymmetric reduction of **1** to yield (*S*)‐**2** and system **HB2** formed by ω‐transaminase (commercial source; ω‐TA) and PLP to catalyze the kinetic resolution of ***rac*** **‐3** through S‐selective deamination. We immobilized the main enzymes (ω‐TA or Tt‐ADH2) on agarose microbeads activated with aldehydes (Ag‐G),^16^ followed by a polymeric coating with PEI through the same amine–aldehyde chemistry,^17^ and a final reduction step to turn the reversible PEI/protein‐agarose imines into irreversible secondary amines (Figure 2 A, step 1). Unfortunately, aldehyde immobilization chemistry inactivated Cb‐FDH (recycling enzyme) at the assayed conditions;^18^ thereby this enzyme was co‐immobilized by ionic adsorption on the PEI‐bed and subsequently cross‐linked with 1,4‐butanediol diglycidyl ether to assure its irreversible attachment (Supporting Information, Table S4; Figure 2 A, step 2).

**Figure 2:**
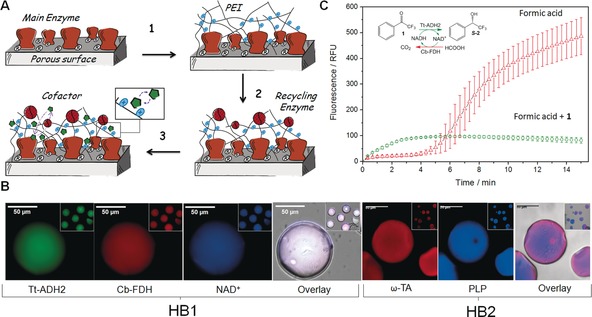
A) Architecture of the self‐sufficient heterogeneous biocatalyst. Cofactor association/dissociation equilibrium is depicted in the inset. B) Spatial distribution of the self‐sufficient heterogeneous biocatalysts (HB1 and HB2) by using fluorescence microscopy. Tt‐ADH2 is labeled with fluoresceine, Cb‐FDH and ω‐TA are labeled with rhodamine, and NAD^+^ and PLP present autofluorescence. C) In operando analysis of the NADH production within a single microbead of HB1 during the redox biotransformation with (green ○) and without NAD^+^ recycling (red ▵). The reaction was monitored and the average fluorescence was quantified by measuring the autofluorescence of NADH at 460 nm in 10 microbeads.

**Table 1:** Catalytic efficiency and reuse of different heterogeneous biocatalysts with soluble and immobilized cofactors.

Enzymes and reactions	Specific activity [μmol mg^−1^ min^−1^]^[d]^	Yield [%]^[d]^	TOF [min^−1^]	TTN [mol product mol NAD^+−1^]^[e]^
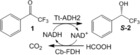	1.76±0.36^[a]^	100^[a]^	0.11^[a]^	10^[a]^
	1.19±0.25^[b]^	100^[b]^	0.079^[b]^	10^[b]^
	0.94±0.17^[c]^	100^[c]^	0.064^[c]^	40^[c]^
				
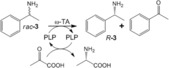	0.046±0.018^[a]^	50^[a]^	0.039^[a]^	5.0^[a]^
	0.005±0.002^[b]^	41^[b]^	0.012^[b]^	3.9^[b]^
	0.030±0.014^[c]^	46^[c]^	0.032^[c]^	16.8^[c]^

[a] Soluble enzymes and cofactor. [b] Soluble cofactor and immobilized enzymes. [c] Co‐immobilized enzymes and cofactor. [d] Specific activity (TtADH2 or ω‐TA) and yield in the first cycle. [e] TTN after 1 cycle for [a,b] and 4 batch cycles for [c]. TOF was calculated as μmol of product per μmol of cofactor in one hour. For reaction conditions, see the Supporting Information, Figures S7–S9 and S11).

Finally, the corresponding cofactor is adsorbed to the cationic bed upon the enzyme immobilization and cross‐linking (Figure 2 A, step 3). In this architecture, both enzymes and PEI are irreversibly bound to the agarose microbeads leaving the enzymes surrounded by the cationic polymer with the ionically adsorbed cofactor (Figure 2 A). The different enzymes were immobilized with yields ranging 79 to 100 % and recovering 14–100 % of their specific activities upon the immobilization process (Supporting Information, Table S4). The co‐immobilization of the cofactors (PLP and NAD^+^) with the enzymes negligible affected their lixiviation pattern (Supporting Information, Figure S5). We then studied the spatial distribution of both autofluorescent cofactors and fluorophore‐labelled enzymes for HB1 and HB2 (Figure 2 B). In all cases, both enzymes and cofactors were homogeneously co‐localized across the porous surface of the agarose microbeads which favors the cofactor shuttling between the enzymes (Figure 2 B; Supporting Information, Figure S6). To demonstrate that the ionically adsorbed cofactors are recycled within the solid phase and remain inside the porous structure, we run the asymmetric reduction of **1** catalyzed by HB1 and monitoring in operando the autofluorescence of NADH within single‐agarose microbeads under the microscope (Figure 2 C). The in operando experiments demonstrate that the immobilized redox cofactors are available for the immobilized enzymes and remain inside the porous microbeads during the reaction. When **1** and formic acid were present, the NADH was not accumulated inside the particle as occurred in the reaction only using formic acid. Then, we evaluated HB1 and HB2 as self‐sufficient heterogeneous biocatalysts able to catalyze their corresponding reactions without addition of exogenous cofactors (Table 1). In batch reactions, the immobilized Tt‐ADH2 and ω‐TA presented lower specific activities than their soluble counterparts.

In the asymmetric reduction of **1**, HB1 exhibited a similar conversion and reaction rate to the co‐immobilized Tt‐ADH2 and Cb‐FDH with exogenous NAD^+^ (Supporting Information, Figure S7). These reactions were run with 2 equivalents of formic acid and 0.1 equivalents of either immobilized or soluble NAD^+^ to enable the cofactor regeneration. A larger excess of sodium formate (100 mm, 10 equivalents) reduced the conversion (<10 %) because of the cofactor lixiviation owing to the weakening of the ionic interactions (Supporting Information, Figure S2 B). The next step was testing the reusability of this self‐sufficient heterogeneous biocatalyst without adding exogenous NAD^+^. HB1 was re‐used for 4 batch cycles, achieving the maximum conversion in each cycle and accumulating a total turnover number (TTN) of 40 (the theoretical maximum; Supporting Information, Figure S8). Likewise, HB2 catalyzed asymmetric deamination of ***rac*** **‐3** without exogenous addition of PLP and surprisingly the reaction was 8‐fold faster with a conversion 1.1‐fold higher than when exogenous PLP was added (Table 1; Supporting Information, Figure S9). When HB2 was loaded with >20 μmol_PLP_ g^−1^, the cofactor seems to be more catalytically available for the immobilized ω‐TA than the soluble PLP whose diffusion across the cationic bed might be hindered owing to the strong PLP‐PEI interactions (Supporting Information, Figure S10). Finally, HB2 was re‐used for up to 4 cycles accumulating a TTN of 16.8 when the theoretical maximum accumulated TTN should be 40. This lower accumulated TTN value was due to the operational inactivation of immobilized ω‐TA; similar results were obtained when reusing the same immobilized enzyme using exogenous PLP (Supporting Information, Figure S11), indicating that the immobilization of the ω‐transaminase must be optimized. Data from the two systems support that the cofactor immobilization is a dynamic process where cofactor molecules shift between the associated and dissociate state inside the porous surface enabling both the regeneration and the reutilization of the cofactors.

The excellent batch performance of immobilized NAD^+^ motivated us to convert **1** into ***S*** **‐2** in a flow reactor without addition of exogenous cofactor. We packed HB1 into a column and fed the reactor with the reaction mixture at different flow rates (10–200) μL min^−1^ (Supporting Information, Table S5). 50 μL min^−1^ was the optimal flow to achieve 100 % conversion with a productivity of 250 μm min^−1^. Under such conditions, the reactor was running for 92 hours achieving a conversion of over 90 % that decayed to 79 % after 107 hours of continuous operation. According to these data, the immobilized NAD^+^ was efficiently re‐cycled, accumulating a TTN of 85 after 107 hours (356 operational volumes) without significant NAD^+^ lixiviation (Figure 3). Additionally, the Tt‐ADH2 in HB1 maintained its high enantioselectivity during the entire biotransformation (*ee* >99 % of ***S*** **‐2**), thus facilitating the work‐up for the isolation of the enantiopure product. We isolated 31.4 mg of ***S*** **‐2** and confirmed its enantiopurity by chiral‐phase HPLC (Supporting Information, Figure S13) and its purity and chemical structure by NMR (Supporting Information, Figure S14). Finally, we successfully reloaded the heterogeneous biocatalyst with fresh cofactor recovering the maximum enzyme performance for three reloading cycles (Supporting Information, Table S6).

**Figure 3:**
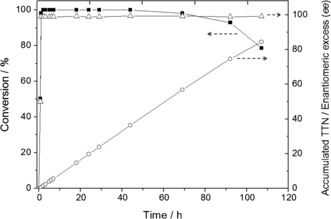
Continuous flow asymmetric reduction of **1** to ***S*** **‐2** catalyzed by HB1 in a packed bed reactor. Conversion of **1** into ***S*** **‐2** (▪), accumulated TTN along the time (○), enantiomeric excess (▵). The bioreactor was operated at 50 μL min^−1^ with 5 mm of **1**, 10 mm of formic acid, acetonitrile 5 % and 10 mm Tris‐HCl solution at pH 7 and 25 °C. During the whole process, less than 10 % NAD^+^ lixiviation was observed (data not shown).

In summary, we have introduced the successful fabrication of self‐sufficient heterogeneous biocatalysts integrating enzymes and phosphorylated cofactors within the same porous microbead through an innovative architecture where the enzymes and PEI are irreversibly bound to the solid surface, and the negative charged cofactors are reversibly adsorbed to the PEI through ion‐exchange interactions. To the best of our knowledge, we have successfully applied a heterogeneous biocatalyst integrating NAD^+^ to asymmetric reduction of ketones in continuous for first time. This pioneer work contributes to one of the challenges of the modern biocatalysis; the cofactor‐free biotransformations mediated by self‐sufficient artificial metabolic cells without genomic regulations.

## Conflict of interest

The authors declare no conflict of interest.

## Supporting information

As a service to our authors and readers, this journal provides supporting information supplied by the authors. Such materials are peer reviewed and may be re‐organized for online delivery, but are not copy‐edited or typeset. Technical support issues arising from supporting information (other than missing files) should be addressed to the authors.

Supplementary
