# Supplementary material for: Co‐immobilized Phosphorylated Cofactors and Enzymes as Self‐Sufficient Heterogeneous Biocatalysts for Chemical Processes
Source: Angew Chem Int Ed Engl. 2016 Dec 21;56(3):771–5. doi: 10.1002/anie.201609758 (PMC5299495; doi:10.1002/anie.201609758)
Supplement: Supplementary file 1 — Supplementary [file ANIE-56-771-s001.pdf]

Angewandte Chemie
Eine Zeitschrift der Gesellschaft Deutscher Chemiker

Supporting Information

Co-immobilized Phosphorylated Cofactors and Enzymes as Self-Sufficient Heterogeneous Biocatalysts for Chemical Processes

*Susana Velasco-Lozano, Ana I. Benítez-Mateos, and Fernando López-Gallego**

anie_201609758_sm_miscellaneous_information.pdf

Supporting Information

Content

List of abbreviations S3

Materials and equipment S4

Fabrication of agarose microbeads coated with polyethyleneimine. S4

Cofactor immobilization S5

Figure S1. Effect of the initial NAD^+ loading............ S6

Calculations of the apparent dissociation constant of the immobilized cofactors S7

Table S2. Apparent dissociation constants of different phosphorylated cofactor ionically absorbed to Ag-GPEI............ S8

Enzyme production and purification............ S8

Figure S3. The SDS-PAGEs of the purification of the enzymes S10

Enzyme activity S10

Cb-FDH kinetic parameters S11

Table S3. Cofactor catalytic availability of soluble and immobilized Cb-FDH with free (166 µM in bulk) and immobilized NAD^+ S12

Figure S4. Kinetic parameters of soluble and immobilized Cb-FDH with soluble NAD^+ S12

Enzyme immobilization............ S12

Table S4. Immobilization yields and expressed activities of immobilized Tt-ADH2, Cb-FDH and ω-TA on Ag-GPEI S14

Fluorescamin- or rodamine-labeling of enzymes S14

In operando *monitoring of cofactor localization during its enzymatic using and regeneration* S14

Figure S5. Co-immobilized phosphorylated cofactors and enzymes on Ag-GPEI............ S15

Figure S6. Confocal microscopy images of agarose beads of HB1 without NAD^+............ S15

Asymmetric reduction of 1 into S-*2 catalyzed by the HB1* S16

Figure S7. Reaction course of the asymmetric reduction of 1 to *S*-2 catalyzed by HB1 or Tt-ADH2 and Cb-FDH biocatalysts S17

Figure S8. Asymmetric reduction of 1 into *S*-2 .... S17

*Deamination of racemic methylbencylamine (*rac-*3) to acetophenone (4) catalyzed by HB2*............ S18

Figure S10. Effect of different loadings of immobilized PLP during the deamination of *rac*-3 into 4 catalyzed by HB2 .... S19

Figure S11. Deamination of *rac*-3 into 4 .... S20

Asymmetric reduction of 1 in flow S20

Figure S12. Scheme of the continuous bioreactor with a packed heterogeneous biocatalyst S21

Figure S13. Chromatographs of standards of *rac*-2 and *R*-2, and the isolated product from the reactor (*S*-2) S21

Figure S14. ^{1}H-NMR and ^{13}C-NMR spectra of produced and purified *S*-2. .... S22

Table S5. Effect of flow rate in the productivity of continuous fed packed reactor of HB1 in the asymmetric reduction of 1 to *S*-2 .... S23

Table S6. Reloading of the heterogeneous biocatalysts with NADH for several cycles. S23

REFERENCES S23

List of abbreviations

1	2,2,2-trifluoroacetophenone
4	acetophenone
Ag	Agarose microbeads
Ag-DVSPEI	Agarose microbeads activated with divinyl-sulfone and further covalent PEI coating
Ag-G	Agarose microbeads activated with glyoxyl gropus
Ag-GPEI	Agarose microbeads activated with glyoxyl gropus further covalent PEI coating
Ag-GTEA	Agarose microbeads activated with triethyl amine groups
Cb-FDH	Formate dehydrogenase from *Candida boidinii*
ee	Enantiomeric excess
FAD^+	Flavin adenine dinucleotide
HB1	Heterogeneous biocatalyst 1, composed by Tt-ADH2, Cb-FDH and NAD^+
HB2	Heterogeneous biocatalyst 2, composed by ω-TA and PLP
K_d	Dissociation constant
K_d^{app}	Apparent dissociation constant
K_M	Michaelis-Menten constant
NAD(P)H	Nicotinamide adenine dinucleotide phosphate, reduced
NAD^+	Nicotinamide adenine dinucleotide
NADH	Nicotinamide adenine dinucleotide, reduced
NMR	Nuclear magnetic resonance
PEI	Polyethyleneimine
PLP	Pyridoxal phosphate
Purolite-GPEI	Purolite microbeads activated with glyoxyl groups and further covalent PEI coating
rac-2	racemic (trifluoromethyl)bencylalcohol
rac-3	racemic methylbencylamine
R-3	*R*-methylbencylamine
S-2	*S*-(trifluoromethyl)bencylalcohol
TOF	Turnover frequency
Tt-ADH2	Alcohol dehydrogenase 2 from *Thermus thermophilus*
TTN	Total turnover number
ω-TA	ω-transaminase
FITC	Fluorescamine isothiocyanate
BDE	1,4-butanediol diglycidyl ether
IPTG	1-thio-β-d-galactorpyranoside

EXPERIMENTAL SECTION

Materials and equipment

Substrates and reagents as 2,2,2-trifluoroacetophenone (**1**), racemic (trifluoromethyl)bencylalcohol (***rac*-2**), *S*-(trifluoromethyl)bencylalcohol 99% (***S*-2**), racemic methylbencylamine (***rac*-3**), acetophenone (**4**), flavin-adenine-dinucleotide sodium salt (FAD^+), pyridoxal 5′-phosphate monohydrate >97% (PLP), sodium formate, fluorescamine isothiocyanate (FITC), rhodamine B isothiocyanate, and polyethyleneimine 25 kDa (PEI) were acquired from Sigma-Aldrich (St. Louis, IL). Nicotinamide-adenine-dinucleotide sodium salt (NAD^+) was purchased from GERBU Biotechnik GmbH (Wieblingen, Germany). Cross-linking diepoxide, 1,4-butanediol diglycidyl ether (BDE), was purchased from Thermo Scientific-Alfa Aesar (Karlsruhe, Germany). 6BCL agarose beads activated with glyoxyl groups (Ag-G) was prepared as described elsewhere, following the same methodology for glyoxyl-activated methacrylate.[1] Epoxy-activated methacrylate beads (ECR8214F) were kindly donated by Purolite (Llantrisant, UK). DVS-activated agarose beads were prepared as previous described (Ag-DVS).[2] ω-Transaminase evo-1.2.129.S (ω-TA) was purchased from Evocatal (Rhein, Germany). All other reagents and solvents were analytical grade or superior.

Fabrication of agarose microbeads coated with polyethyleneimine.

Coated Ag-G beads with PEI (Ag-GPEI) were prepared by mixing a suspension (1:10 p/v) of Ag-G and PEI (25 kDa, at final concentration of 10 mg/mL) in bicarbonate buffer solution 100 mM pH 10 under orbital agitation for 1 h at 25 ºC. Later, solid sodium borohydride was added (1 mg/mL) and incubated 30 min at 25 ºC. Finally, the Ag-GPEI was washed 5 times with 10 volumes of water and stored at 4 ºC. The same protocol was followed to coat both Purolite and Ag-DVS materials but skipping the reduction

step since the PEI irreversibly and directly reacts with the epoxy and vinyl groups in the surface of Purolite and Ag-G, respectively. The supports modified with PEI were characterized by using N-succinimidyl-3-(2-pyridyldithio)-propionate that specifically reacts with the primary amines and the product can be colorimetrically quantified at 343 nm.[3]

Cofactor immobilization

Ionic immobilization of phosphorylated cofactors was achieved by incubating 10 volumes of soluble cofactor at the indicated concentration in 10 mM sodium phosphate at pH 7 with 1 volume of solid agarose beads (with or without previously immobilized enzymes). The suspension was kept under orbital agitation at 25 rpm for 1 h at 4 ºC and finally filtered and washed two times with 1 volume of 10 mM sodium phosphate at pH 7. Concentration of the immobilized cofactors were calculated by measuring the absorbance of the supernatants after the adsorption process and after each wash at the proper wavelength (260 nm for NAD^+, 345 nm for NADH, 450 nm for FAD^+ and 390 nm for PLP) in a Varioskan™ Flash Multimode Reader (Thermo Scientific). In all cases, the cofactors were washed for several cycles after the immobilization step. Each cycle was carried out by adding 10 volumes of sodium phosphate buffered solution at 10 mM pH 7 per gram of immobilized cofactor.

Table S1. Effect of the pH and chemistry bound in the immobilization of NAD^+.

	NAD^+ (µmol/g of support)			
	Ag-GTEA		Ag-GPEI	
Washing number	pH 7	pH 9	pH 7	pH 9
0	11.2	18.4	17.5	18.2
1	0	1.9	6.7	3.0
2	0	0	4.9	1.0
3	0	0	4.4	0.4
4	0	0	4.0	0.2
5	0	0	3.8	0.1

Washing steps were performed by adding 10 volumes of sodium phosphate or carbonate 10 mM at pH 7 or 9 respectively.

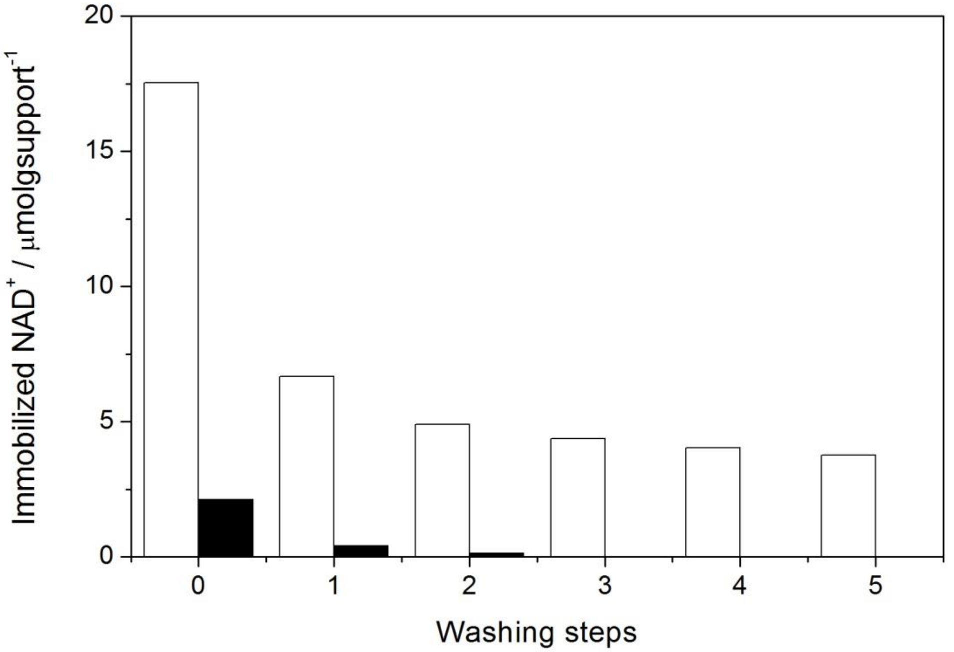

Figure S1. Effect of the initial NAD^+ loading (white bars: 10 mM and black bars 1 mM) during its immobilization on Ag-GPEI. In all cases washing steps were carried out by adding 10 volumes of sodium phosphate buffered solution at 10 mM pH 7.

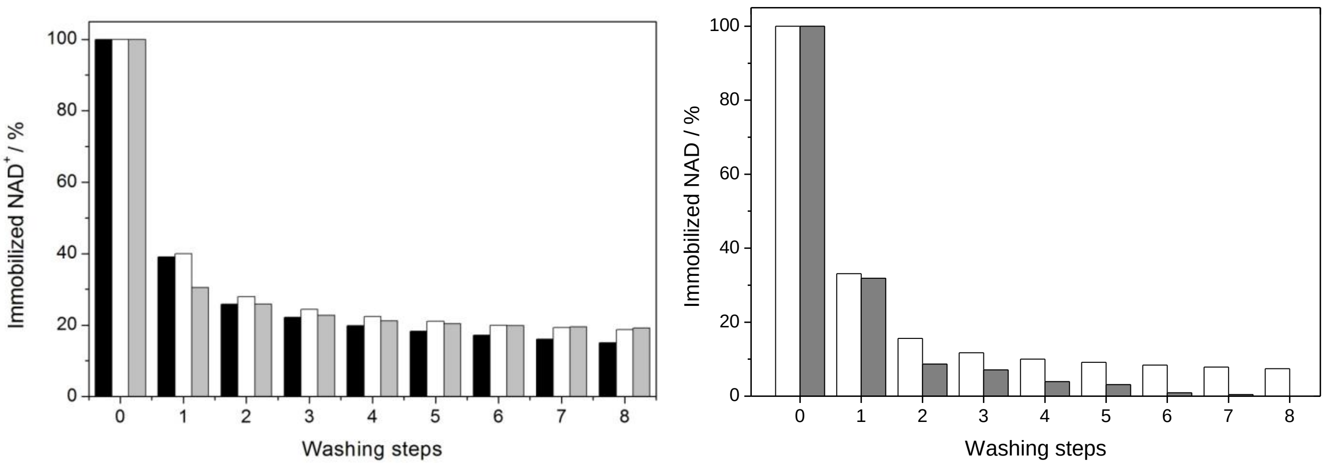

Figure S2. (A) Immobilization of NAD^+ on different PEI-coated supports. Ag-GPEI (black bars), Purolite-GPEI (white bars) and Ag-DVSPEI (grey-bars). In all cases washing steps were carried out by adding 10 volumes of sodium phosphate buffered solution at 10 mM pH 7. The initial immobilized NAD^+ in each case corresponds to 24, 23 and 22 µmol of NAD^+ per gram of support for initial load of 10 mM, respectively. (B) Effect of ionic strength on the residual immobilized NAD^+ of HB1 after washing treatments at different formate concentrations. In all cases washing steps were carried out by adding 10 volumes of formic acid 10 mM (white bars) and 100 mM (grey bars) in sodium phosphate buffered solution at 10 mM pH 7.

Calculations of the apparent dissociation constant of the immobilized cofactors

An apparent dissociation constant (K_d^{app}) is defined for the interaction between the PEI and the different cofactors. Assuming that the association/dissociation equilibrium is defined by equation (1) based on the ionic interaction of one molecule of negatively charged cofactor with one positively amine group of the PEI.

$$[N^{(+)}\text{-}Cof^{(-)}] \leftrightarrow [N^{(+)}] + [Cof^{(-)}]_{free} \qquad (1)$$

$$[N^{(+)}\text{-}Cof^{(-)}] = [Cof^{(-)}]_{bound} \qquad (2)$$

$[Cof^{(-)}]_{free}$ are the cofactor molecules not bound to 1 g of Ag-GPEI after the adsorption process (experimentally determined, table S2), $[Cof^{(-)}]_{bound}$ is defined as the cofactor molecules bound per gram of Ag-GPEI after the adsorption process (experimentally determined, Fig. 1A) and $[N^{(+)}]$ is the number of free positive charges per gram of Ag-GPEI microbeads after the adsorption process. $[N^{(+)}]$ can be calculated according to the equation (3), where $[N^{(+)}]_o$ are the total positives charges of PEI before the incubation with the cofactors.

$$[N^{(+)}]=[N^{(+)}]_o - [Cof^{(-)}]_{bound} \qquad (3)$$

$[N^{(+)}]_o$ can be calculated assuming that 1 gram of Ag-GPEI contains 4 μmol_{PEI}/g that means 1724 $\mu mol_{monomer\ (C2N2H6)}$/g following our activation protocol and using PEI 25 kDa. Based on a positive charge density of $[N^+]/C=0.207$ calculated by Kim *et al.*[4] using XPS, we can assume that 1 monomer of the cationic polymer contains 2 C atoms and 0.414 positive charges, then $\mathbf{[N^{(+)}]_o=714\ \mu mol/g}$. With the

experimental data of the bound and free cofactor together with the assumptions to determine $[N^{(+)}]_o$, we can calculate $[N^{(+)}]$ and define the apparent dissociation constant (K_d^{app}) as equation (4):

$$K_d^{app}=([N^{(+)}]_o - [Cof^{(-)}]_{bound}) \times [Cof^{(-)}]_{free})/ [Cof^{(-)}]_{bound} \qquad (4)$$

Table S2. Apparent dissociation constants of different phosphorylated cofactor ionically absorbed to Ag-GPEI

Cofactor	$[Cof^{(-)}]_{bound}$ (μmol/g)[a]	$[Cof^{(-)}]_{free}$ (μmol/g)[a]	K_d^{app} (μmol/g)[b]
NAD^+	17.5	82.5	3283
PLP	83.3	16.7	126
FAD^+	46.1	53.9	781

[a]The free and bound cofactors were calculated by incubating 1 mL of 10 mM cofactor in 10 mM sodium phosphate at pH 7 with 0.1 g of Ag-GPEI for 60 minutes when the concentration of the free cofactor is constant (equilibrium in batch). [b]The K_d^{app} was calculated according to equation 4.

Enzyme production and purification

Alcohol dehydrogenase (Tt-ADH2)[5] from *T. thermophilus,* formate dehydrogenase from *Candida boidinii* (Cb-FDH) were overexpressed in *Escherichia coli* as described elsewhere.[6] Briefly, a total of 1 mL of an overnight culture of *E. coli* BL21 transformed with the respective plasmid (pET22-Tt-ADH2, or pET28b-Cb-FDH) was inoculated in a 50 mL of Luria-Bertani (LB) broth containing kanamycin (final concentration of 30 μg mL^{-1}) for pET28b-Cb-FDH and ampicillin (100 μg mL^{-1}) for pET22-Tt-ADH2. For pET22-Tt-ADH2 the resulting culture was incubated at 37°C with energetic shaking until the OD_{600nm} reached 0.6. At that point, the culture was induced with 1 mM of 1-thio-β-d-galactorpyranoside (IPTG). Cells were grown at 37°C for 3 h and then harvested by centrifugation at 1290 *g* during 30 min at 4 ºC. For pET28b-Cb-FDH, the resulting culture was incubated at 21°C with

energetic shaking until the OD_{600nm} reached 0.6, and then the culture was induced with 1 mM IPTG. Cells were grown at 21°C for 18 h and then harvested by centrifugation at 1290 *g* during 30 min at 4 ºC. *Purification.* Cb-FDH was as follows: the resulting pellet was resuspended in one-tenth of its original volume of 10 mM sodium phosphate buffer solution (pH=7, binding buffer). Cells were broken by sonication (LABSONIC P, Sartorius Stedim biotech) at amplitude=30% and cycle=1 during 5 min. The suspension was centrifuged at 10528 *g* during 30 min at 4 ºC. The supernatant containing the enzyme was collected and passed through a Talon resin equilibrated with binding buffer. Following the protein binding to the column, the column was washed three times with binding buffer prior to the protein elution with elution buffer (binding buffer supplemented with 300 mM imidazole). The eluted protein was gel-filtered by using PD-10 columns (GE healthcare) to remove the imidazole and exchange the enzyme buffer to 10 mM sodium phosphate pH 7. Tt-ADH2 was as follows: the cells were broken by sonication (LABSONIC P, Sartorius Stedim biotech) at amplitude=30% and cycle=1 during 5 min. The suspension was centrifuged at 10528 *g* during 30 min at 4 ºC. The supernatant containing the enzyme was incubated at 70 ºC for 1 h to remove all mesophilic proteins, the thermophilic one remain at the supernatant that is separated by centrifugation at 10528 *g* during 30 min at 4 ºC. SDS-PAGE and Bradford assay were carried out after each production to determine the purity, concentration and specific activity of the enzymes.

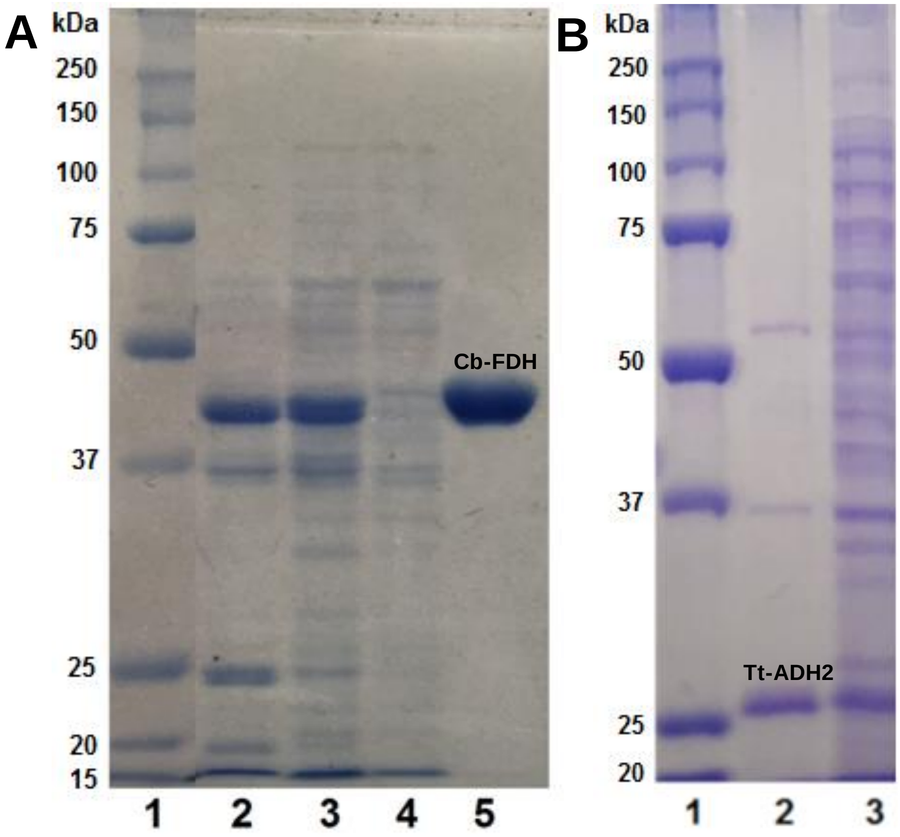

Figure S3. The SDS-PAGEs of the purification of the enzymes. A: Cb-FDH and B:Tt-ADH2. 1: Molecular weight markers (BioRad Precision Plus Protein All Blue Standard), 2A: Crude extract, 3A: Soluble fraction after cell disruption by sonication, 4A: Elution fraction, 5A: pure Cb-FDH (band 40 kDa), 2B: pure Tt-ADH2 (band 27 kDa) and 3B: Soluble fraction after cell disruption by sonication.

The final protein preparations had 4.96 mg/mL and 0.6 mg/mL (determined with Bradford assay[7]) for Cb-FDH and Tt-ADH2, respectively; and specific activities of 2.0 U/mg for Cb-FDH (determined spectrophotometrically in the oxidation of NAD^+ in the presence of formate) and 9.92 U/mg for Tt-ADH2 (determined spectrophotometrically with **1** as substrate).

Enzyme activity

The enzymatic activities were spectrophotometrically measured in 96-well plates by monitoring the absorbance at the corresponding wavelenght in a Varioskan™ Flash Multimode Reader (Thermo Scientific). For both soluble and immobilzed enzyme the activity were measure under orbital shaking

integrated into the reader. ***Tt-ADH2* activity.** 200 µL of a reaction mixture containing 10 mM of **1**, 0.25 mM of NADH in sodium phosphate buffer (pH 7) were incubated with 5 µL of enzymatic solution or suspension at 25 ºC. The decrease in the absorbance was monitored at 340 nm. One unit of activity was defined as the amount of enzyme that was required to either reduce or oxidize 1 µmol of the corresponding nicotinamide cofactor at 25 ºC and the corresponding pH. **FDH activity**. 200 µL of 1 mM NAD^+ and 100 mM formic acid in 25 mM sodium phosphate buffer (pH=7) were incubated with 5 µL of enzymatic solution at 25 ºC. The increase in the absorbance was monitored at 340 nm. One unit of activity was defined as the amount of enzyme that was needed to either reduce or oxidize 1 µmol of the corresponding nicotinamide cofactor at 25 ºC and the corresponding pH. **ω-TA activity.** the activity was determined by recording the increment in the absorbance at 245 nm of 200 µL of a reaction mixture containing ***rac*-3** (2 mM), sodium pyruvate (2 mM), PLP (0.1 mM) in potassium phosphate buffered solution 50 mM pH 8. The reaction was initiated by adding 5 µL of the enzymatic solution or suspension to the reaction mixture. One unit of ω-TA activity was defined as the amount of enzyme required for the production one µmol of acetophenone per minute.

Cb-FDH kinetic parameters

The kinetic parameters (K_M and V_{max}) were determined at pH 7 and 25 ºC for the soluble and the immobilized Cb-FDH following the spectrophotometric method described above and using 5000-50 µM of soluble NAD^+ and 538-95 µM of immobilized NAD^+ on Ag-GPEI (Table S3 and Fig. S4).

Table S3. Cofactor catalytic availability of soluble and immobilized Cb-FDH with free (166 µM in bulk) and immobilized NAD^+ (6.6 µmol/g support = 166 µmol in bulk).

Enzyme form	Cofactor form	Specific activity (U/mg)	NAD^+ available (µM)	NAD^+ available (%)
Soluble	Soluble	1.97±0.005[a]	166	100
	Immobilized	0.19±0.06[b]	5.8±1.9[c]	3.5±1.1
Immobilized	Soluble	0.12±0.01[a]	166	100
	Immobilized	0.13±0.01[b]	177±21.2[c]	107.2±12.8

[a]The specific activities of both soluble and immobilized Cb-FDH were calculated by inserting 166 µM of soluble NAD^+ as substrate concentration in the Michaelis-Menten equation using the corresponding kinetics parameters (Vmax and K_M) experimentally determined (Fig. S4) Ae = (Vmax x 166)/K_M +166); where Ae = specific activity in U/mg, Vmax = maximum specific activity in U/mg and K_M = Michaelis-Menten constant in µM. [b] The specific activities were experimentally determined using 166 µM of immobilized NAD^+. [c] The catalytically available NAD^+ concentration was calculated by inserting the specific activity experimentally determined [b] in the Michaelis-Menten equation using the corresponding kinetics parameters (Vmax and K_M) for the soluble and the immobilized Cb-FDH experimentally determined (Fig. S4). $[NAD^+]$=(Ae x K_M)/(Vmax - Ae), where $[NAD^+]$ is the catalytically available immobilized NAD^+ concentration in µM, Ae = specific activity in U/mg, K_M = Michaelis-Menten constant in µM, Vmax = maximum specific activity in U/mg.

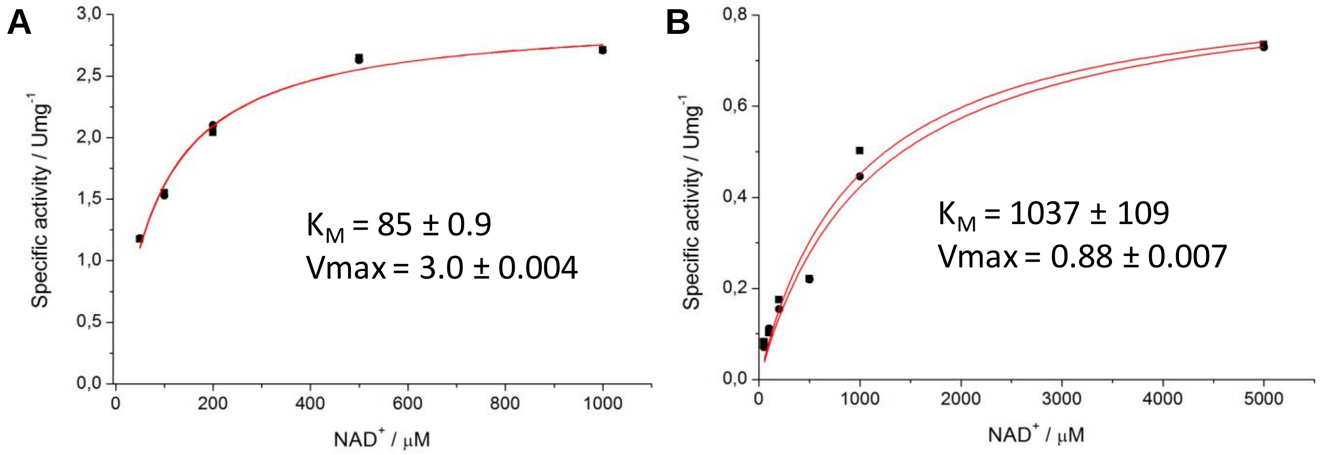

Figure S4. Kinetic parameters of soluble (A) and immobilized (B) Cb-FDH with soluble NAD^+.

Enzyme immobilization

Co-immobilization of Tt-ADH2 and Cb-FDH:[8] 10 volumes of Tt-ADH2 (0.078 mg/mL) in 100 mM sodium bicarbonate buffer at pH 10 were mixed with 1 volume of Ag-G and incubated at room

temperature with orbital shaking for 2 h. Immobilization course was followed spectrophotometrically by measuring the activity of both suspension and supernatant. Later, the suspension was filtered and 10 volumes of a PEI solution (10 mg/mL) in the same buffer pH 10 were added. After 1 h of incubation at room temperature, 1 mg/mL of solid sodium borohydride was added and incubated 30 min at 4 ºC. Then, the suspension was filtered and washed 5 times with 10 volumes of 10 mM sodium phosphate buffer at pH 7. Afterwards, the immobilization of the second enzyme was conducted by adding 10 volumes of Cb-FDH solution (4.5 mg/mL) in sodium phosphate buffered solution 10 mM pH 7 and incubating 1 h at room temperature with orbital shaking. Then, the suspension was filtered and the solid was incubated with 10 volumes of BDE (30 mM at pH 7) for 1 h at room temperature. Finally, the suspension was filtered and washed 2 times with 5 volumes of sodium phosphate buffered solution 10 mM pH 7 and stored at 4 ºC.

Immobilization of ω-TA: 10 volumes of a solution of ω-TA (5 mg of crude powder/mL) in 100 mM sodium bicarbonate buffer at pH 10 were added to 1 volume of Ag-G and incubated for 30 min at room temperature with orbital shaking. Immobilization course was followed spectrophotometrically by measuring the activity in the suspension and in the supernatant. Afterwards, the suspension was filtered and 10 volumes or PEI solution in the same buffer pH 10 (10 mg/mL) were added. After 1 h of incubation at room temperature, 1 mg/mL of solid sodium borohydride was added and incubated 30 min at 4 ºC. Then, the suspension was filtered and washed 5 times with 10 volumes of sodium phosphate buffered solution 10 mM pH 7. Immobilization yields and expressed activity of all the prepared biocatalysts are shown in (Table S4).

Table S4. Immobilization yields and expressed activities of immobilized Tt-ADH2, Cb-FDH and ω-TA on Ag-GPEI**.**

Enzymes	Load (mg/g support)	Immobilization yield (%)[a]	Relative immobilized specific activity(%)[b]	Final expressed activity (U/g support)
Tt-ADH2[*]-Cb-FDH	0.63	79	113	6.15
Tt-ADH2-Cb-FDH[*][c]	43.7	100	52	59.60
ω-TA	43.4	98	14	0.36

[*]Referred enzyme. [a] Immobilization yield (%)=(immobilized protein/offered protein) x 100. [b] Relative immobilized specific activity is defined as the (specific activity of immobilized enzyme/specific activity of soluble enzyme) x 100, where the specific activity of the immobilized enzyme is the final expressed activity/Load. The specific activity of soluble Tt-ADH2 = 8.63 U/mg, soluble Cb-FDH = 2.62 U/mg and soluble ω-TA = 0.059 U/mg. [c] After 5 washes with a mixture of 10 mM of formic acid, 5 mM of **1** and 10 mM sodium phosphate buffer at pH 7 the heterogeneous biocatalyst maintained > 95% of residual Cb-FDH activity; and after a final wash with 0.5 M of NaCl, more than 85% of the Cb-FDH activity was retained in the heterogeneous biocatalyst.

Fluorescamin- or rodamine-labeling of enzymes

Fluorescent labels were done according reported methodology.[9] An enzyme solution in 100 mM of sodium bicarbonate buffered solution at pH 9.5 was mixed (1:20 molar ratio) with fluorescamine isothiocyanate (FITC) or rhodamine B isothiocyanate solution in DMSO (10 mg/mL) and incubated 1 h with gentle agitation at 25 ºC with light protection. Later, the remaining FITC or rhodamine was eliminated by filtering the enzyme solution in a tangential ultrafiltration unit (10 kDa) with a sodium phosphate buffered solution 100 mM sodium carbonate at pH 10 and 10 mM sodium phosphate at pH 7 for Tt-ADH2 and Cb-FDH, respectively, until no coloration was observed in the filtered solution.

In operando *monitoring of cofactor localization during its enzymatic using and regeneration*

Reactions were done in a clear well of a micro-slide (8 well) where 180 µL of a HB1 suspension (in 10 mM of sodium phosphate buffered solution pH 7 [1:20 v/v]) was placed and later the reaction was started by adding 20 µL of substrate solution at a final concentration of 5 mM of **1**, 10 mM of formic acid, 5% acetonitrile in sodium phosphate buffer 10 mM pH 7. The fluorescence monitoring was carried out in a cell axio observer microscope with an excitation wavelength of 365 nm and 402-448 nm of

emission during 15 min at 25 ºC.

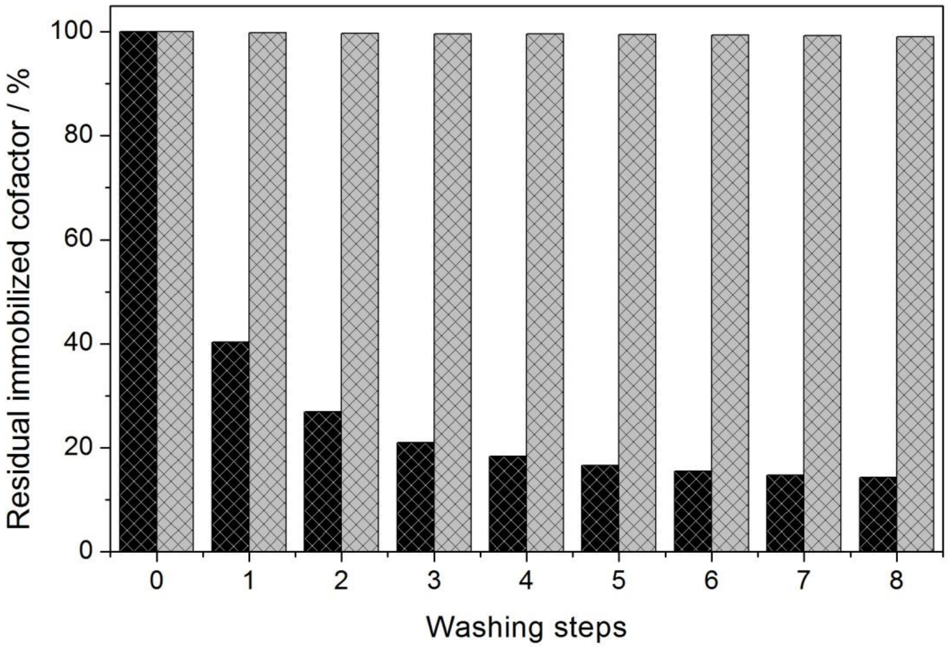

Figure S5. Co-immobilized phosphorylated cofactors and enzymes on Ag-GPEI**.** Co-immobilized NAD^+ and Tt-ADH2-Cb-FDH (grid black bars) and PLP and ω-TA (grid grey bars). The initial immobilized cofactor in each case corresponds to 24 and 21 µmol of NAD^+ and PLP per gram of support, respectively.

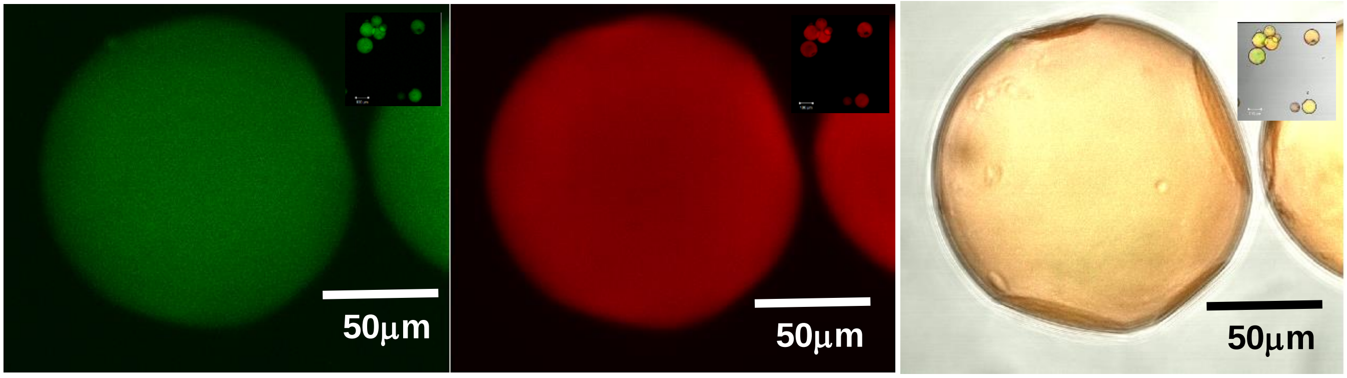

Figure S6. Confocal microscopy images of agarose beads of HB1 without NAD^+**.** Tt-ADH2 labelled with FITC (green) and Cb-FDH labelled with rhodamine (red) were co-immobilized on the same agarose microbeads following the protocol described in the experimental section. Images were taken in the ecuatorial plane demonstrating that both enzymes homogeneously co-localized across the whole porous structure of the agarose microbeads. The right side image overlays the signals of fluoresceine, rhodamine and brightfield.

*Asymmetric reduction of **1** into **S-2** catalyzed by the HB1*

Reaction mixture consisted in 5 mM of **1**, 10 mM sodium formate, 5% (v/v) of acetonitrile in a 10 mM sodium phosphate at pH 7, and 0.5 mM of NAD^+ when exogenous cofactor was added. For batch reactions 1 mL of reaction mixture was added to 50 mg (5%) of solid biocatalyst placed in a micro Bio-spin^{TM} chromatographic column (BIO-RAD) and maintained under gentle rotational agitation (50 rpm) at 25 ºC. The reaction was stopped by vacuum filtration and the heterogeneous biocatalyst was washed with 2 volumes of 10 mM sodium phosphate buffer to be re-used in the next cycle. Reaction curses were followed by withdrawing reaction samples of 5 μL which were properly filtered and diluted in the mobile phase for the UPLC analysis method. The samples were analyzed by Ultra Performance Liquid Chromatograph (UPLC, Waters) in an ACQUITY UPLC BEH C18 column (50 × 2.1 mm). Elution of compounds was carried out at an isocratic flow of 0.3 mL/min with a mobile phase of 0.1% formic acid in water/acetonitrile (65:35). Detection of compounds was done at 254 nm. Retention times were 2.18 min for **1** and 4.0 min for ***rac*-2**. The conversion degree was calculated by fitting the peak's area with a calibration curve under the same elution conditions. Enantiomeric excess (*ee*) of released ***S*-2**, was determined by HPLC (1120 compact LC, Agilent) with a Lux Cellulose-1 column (250 mm × 4.6 mm). Elution of compounds was carried out with an isocratic flow of 1 mL/min using a mobile phase of water/methanol (50/50). Enantiomers were detected at 254 nm with retention times of 17.4 min for ***S*-2** and 22.0 min for ***R*-2.**

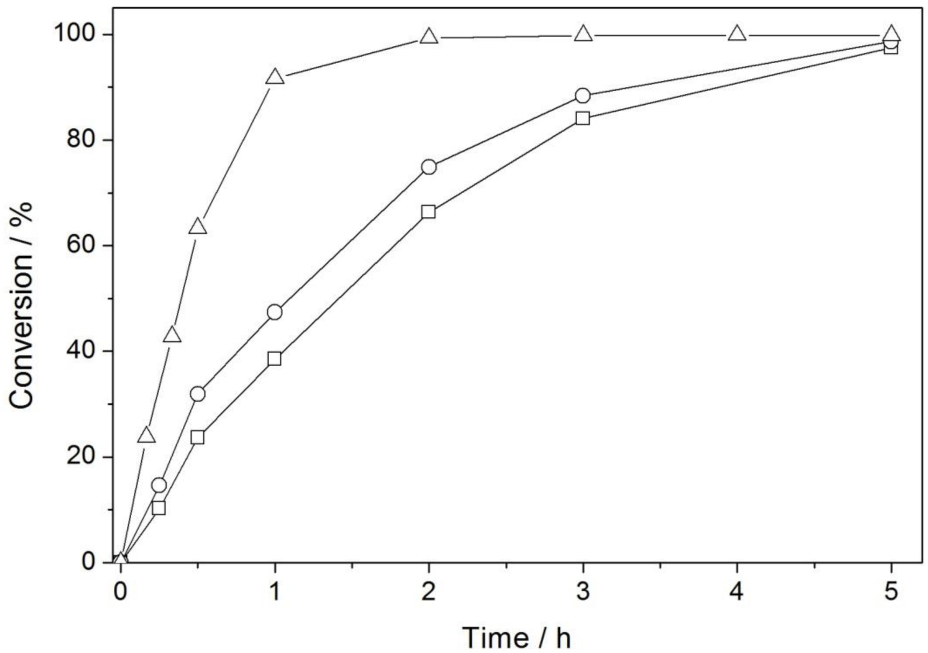

Figure S7. Reaction course of the asymmetric reduction of **1** to ***S*-2** catalyzed by HB1 or Tt-ADH2 and Cb-FDH biocatalysts. Exogenously added cofactor and co-immobilized enzymes (circles), co-immobilized cofactor and enzymes (HB1) (squares), and soluble enzymes and cofactors (triangles). Both reactions were carried out at 5 mM of **1**, 10 mM of formic acid, 0.5 mM of soluble or immobilized cofactor, acetonitrile 5% in phosphate buffered solution 10 mM pH 7 at 25 ºC.

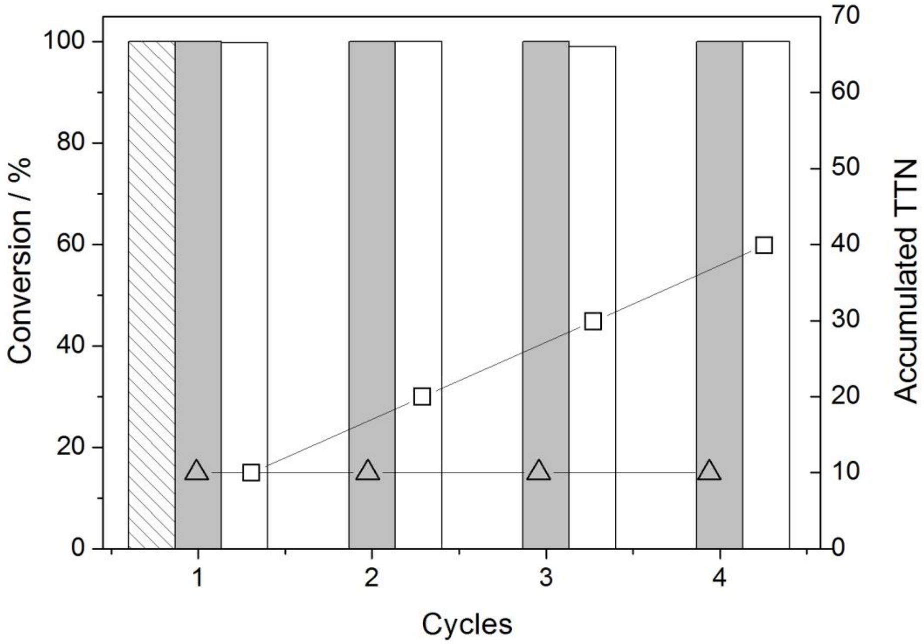

Figure S8. Asymmetric reduction of **1** into ***S*-2** catalyzed by co-immobilzed NAD^+, Tt-ADH2 and Cb-FDH (HB1) (white bars and squares), by co-immobilized Tt-ADH2 and Cb-FDH using soluble NAD^+ (grey bars and triangles) in consecutive batch reactors, and by soluble enzymes and cofactor (dashed white bar). Reactions were carried out at 5 mM of **1** and 0.5 mM of soluble or immobilized NAD^+, 10 mM of formic acid, acetonitrile 5% in phosphate buffered solution 10 mM pH 7 at 25 ºC. Bars correspond to conversion degree, while symbols correspond to the accumulated TTN.

*Deamination of racemic methylbencylamine (***rac-*3*) to acetophenone (*4*) catalyzed by HB2*

Reactions were carried out in a micro Bio-spin™ chromatographic column (BIO-RAD) where 100 mg of solid immobilized biocatalyst was placed. To start the reaction, 1 mL of a reaction mixture composed by 10 mM of ***rac*-3**, 100 mM sodium pyruvate, 5% (v/v) of acetonitrile in a potassium phosphate buffered solution 10 mM pH 8 was added and maintained under gentle rotational agitation (50 rpm). The reaction was stopped by vacuum filtration and the heterogeneous biocatalyst was washed with 2 volumes of 10 mM sodium phosphate buffer to be re-used in the next cycle. Reaction rate and conversion degree was determined by withdrawn filtered reaction samples and analyze them by Ultra Performance Liquid Chromatograph (UPLC, Waters) in an ACQUITY UPLC BEH C18 column (50 × 2.1 mm). Elution of compounds was carried out at constant flow of 0.3 mL/min with the following mobile phase gradient: 95:5 of 0.1% formic acid in water/acetonitrile maintained 2 min, and then up to reach 65:35 in 4 min, and the following 4 min to restore the initial conditions. Detection of compounds was done at 254 nm. Retention times were 2.2 min for ***rac*-3** and 4.7 min for **4**. The conversion degree was calculated by fitting the peak's area with a calibration curve under the same elution conditions.

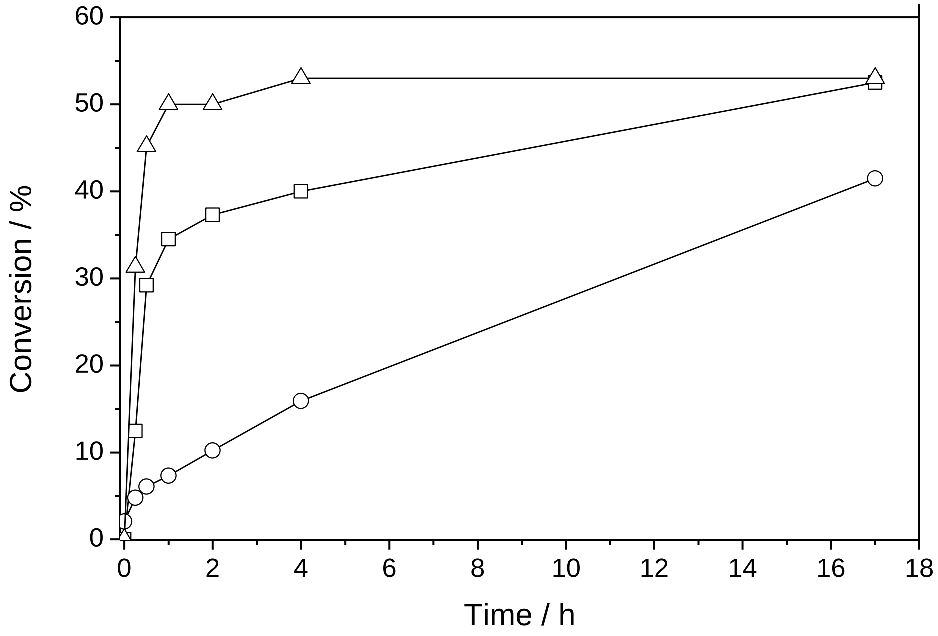

Figure S9. Reaction curse of deamination of ***rac*-3** into **4** in the presence of excess of pyruvate with molar ratios of substrate (10 mM) and cofactor (1 mM) catalyzed by ω-TA. Exogenously added cofactor and co-immobilized enzymes (circles), co-immobilized cofactor and enzymes (HB2) (squares), and soluble enzymes and cofactors (triangles)).

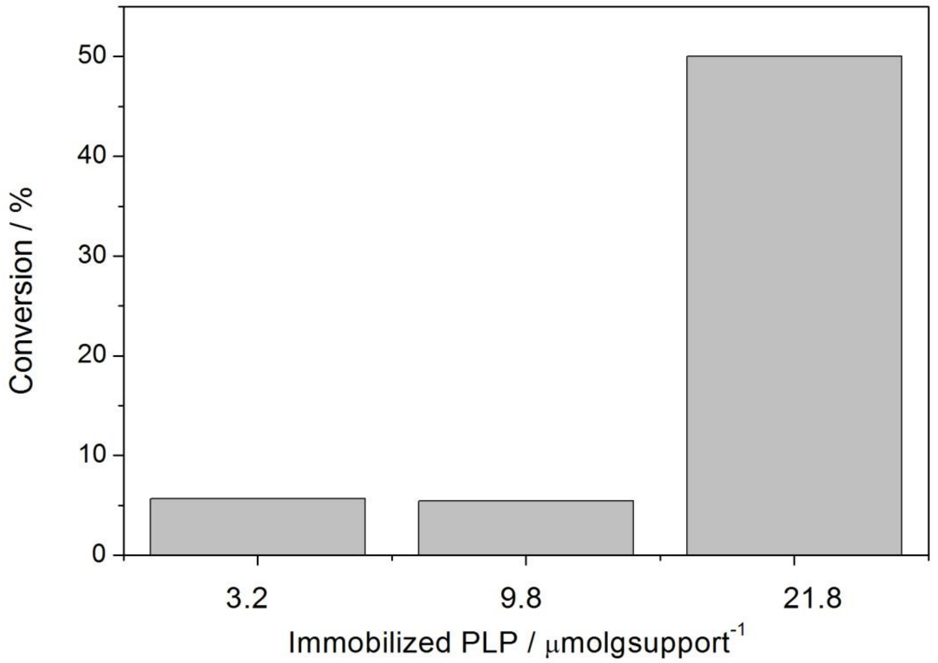

Figure S10. Effect of different loadings of immobilized PLP on the efficiency of the deamination of ***rac*-3** into **4** catalyzed by co-immoiblized PLP and ω-TA (HB2). HB2 loading less than 20 μmol_{PLP}/g reduces the reaction conversion <5%, which points out that the entire pool of immobilized PLP remains adsorbed to the PEI, preventing the cofactor shuttling between the ω-TA active sites due to its low Kdapp (Table S2).

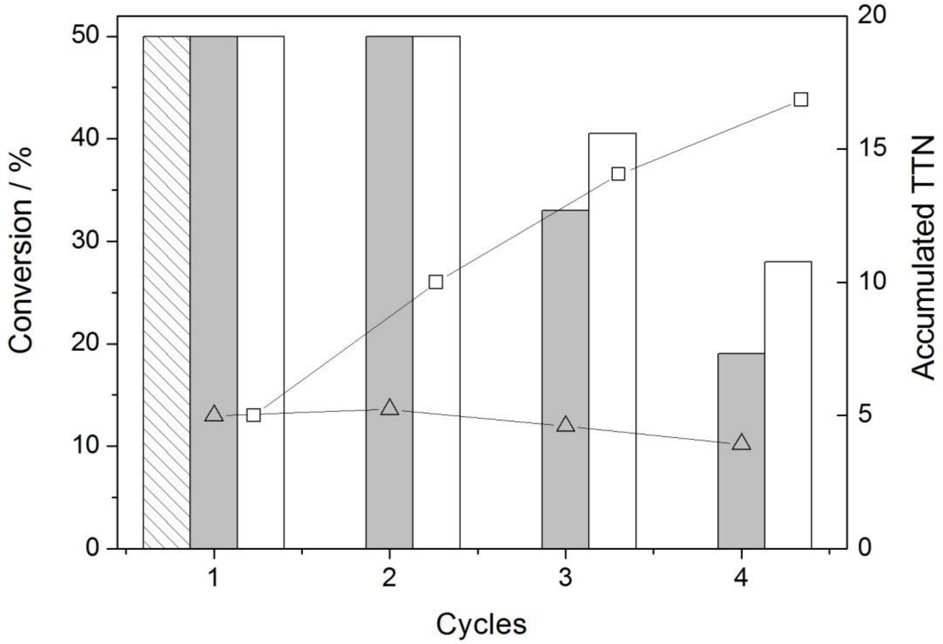

Figure S11. Deamination of ***rac*-3** into **4** in the presence of excess of pyruvate with molar ratios of substrate (10 mM) and cofactor (1 mM) catalyzed by ω-TA. Soluble ω-TA and PLP (dashed white bar), immobilized ω-TA and soluble PLP (grey bars and triangles) and co-immobilzied PLP and ω-TA (HB2) (white bars and squares). Bars correspond to conversion degree, while symbols correspond to the accumulated TTN.

*Asymmetric reduction of **1** in flow*

0.9 g of HB1 (containing 0.023 µmol/g support of Tt-ADH2, 43.7 µmol/g support of Cb-FDH and 20 µmol/g support of NAD^+) were packed into a column (2 x 0.4 cm) and connected to a flow system (Fig. S12). A reaction mixture containing 5 mM of **1**, 10 mM sodium formate, 5% (v/v) of acetonitrile in a 10 mM tris-HCl at pH 7 passed through the column at different flow rates (10-200 µL x min^{-1}). Different samples were collected from the outlet of the system and analyzed by UPLC and chiral HPLC. After 92 hours of running the continuous reactor, we collected 276 mL of product that were further extracted with CH_2Cl_2 (3 x 500 mL). The organic phase was dried with $MgSO_4$ anhydrous and the solvent was evaporated. After vacuum dry, we obtained a yellow and oily product that was analyzed by chiral-HPLC (Fig. S13) to confirm its enantiopurity (>99%) and by NMR (^{1}H-NMR and ^{13}C-NMR) to determine its purity. (***S*-2**) **^{1}H NMR** (500 MHz, $CDCl_3$) δ 7.50 – 7.37 (m, 2H), 7.36 – 7.29 (m, 3H), 4.94 (q, *J* = 6.7

Hz, 1H). **^{13}C NMR** (126 MHz, $CDCl_3$) δ 124.23 (q, $J_{C\text{-}F}$= 282.0 Hz), 72.84 (q, $J_{C\text{-}F}$ = 31.9 Hz) (Fig. S14).

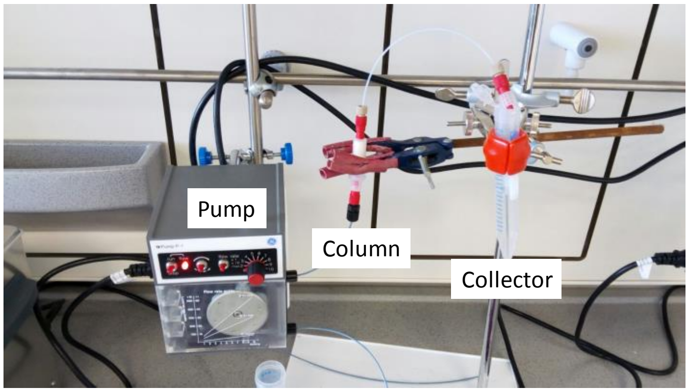

Figure S12. Scheme of the continuous bioreactor with a packed heterogeneous biocatalyst formed by Tt-ADH2, Cb-FDH and NAD^+ to asymmetrically reduce **1**.

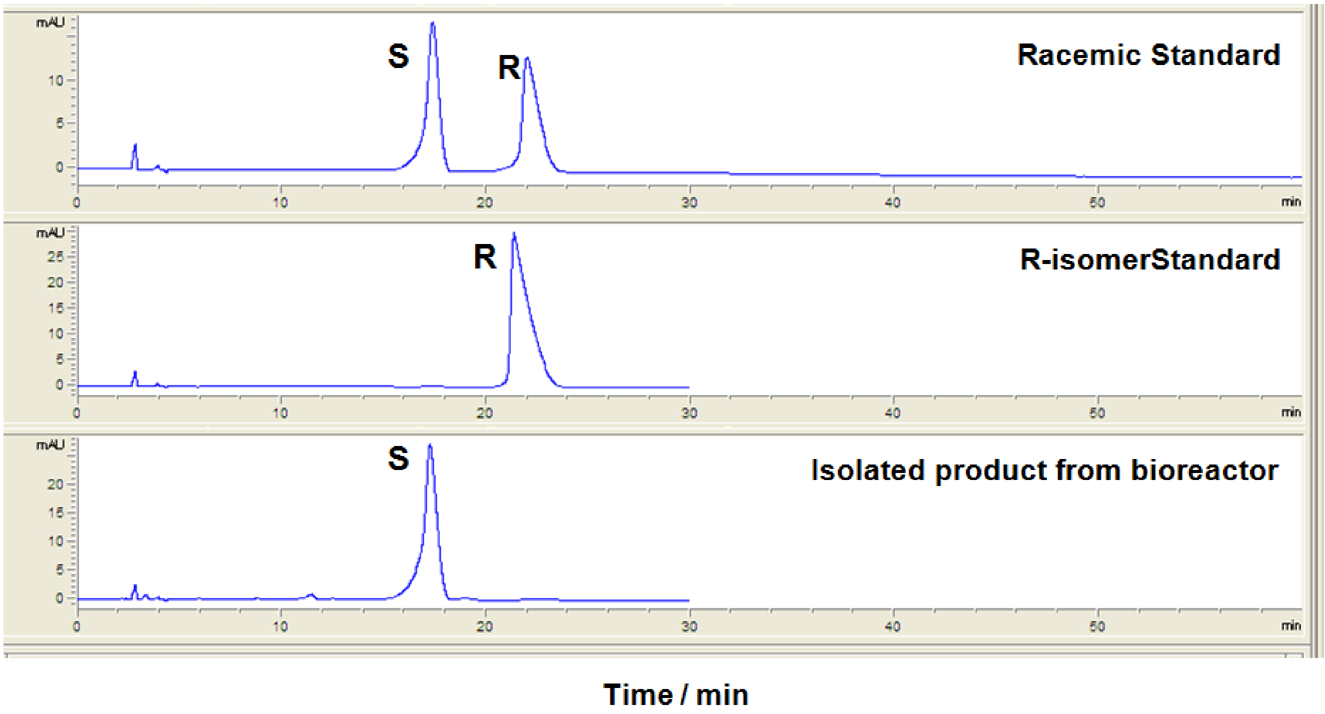

Figure S13. Chromatographs of standards of ***rac*-2** and ***R*-2**, and the isolated product from the reactor (***S*-2**).

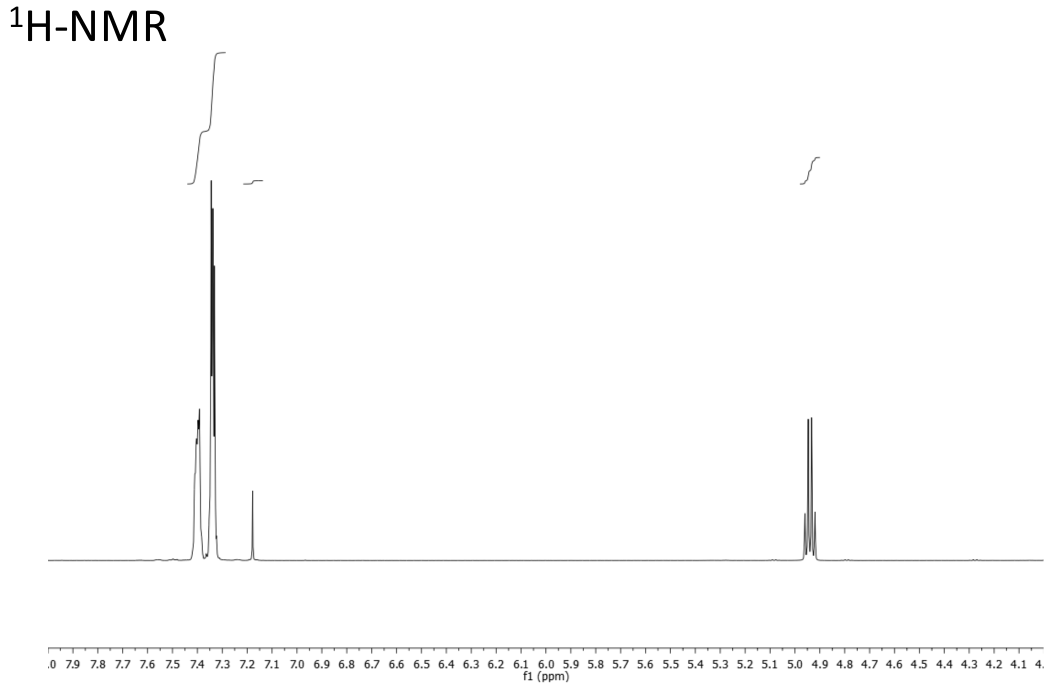

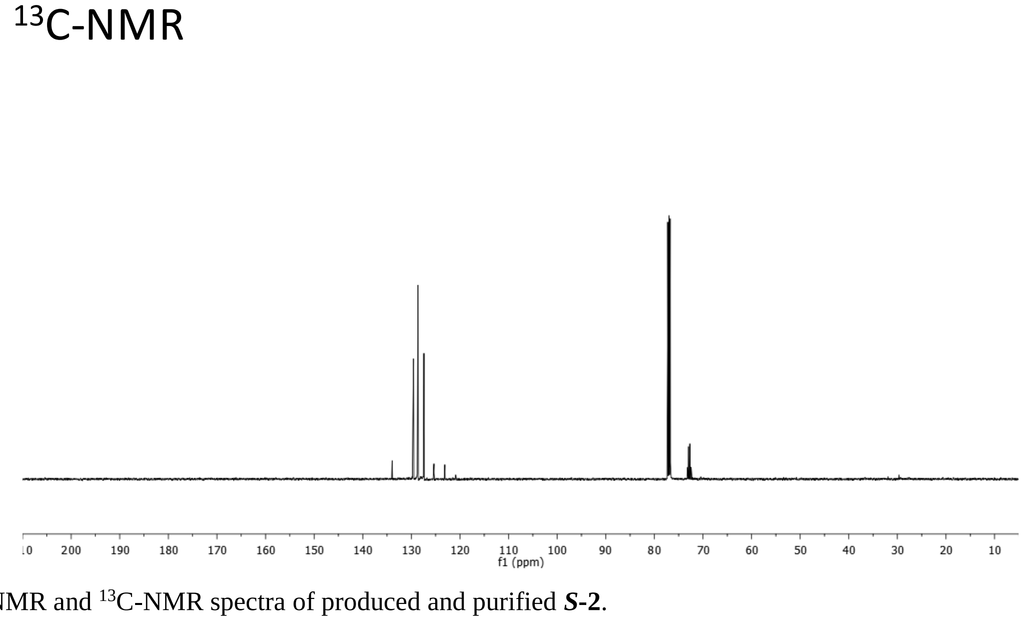

Figure S14.^{1}H-NMR and ^{13}C-NMR spectra of produced and purified ***S*-2**.

Table S5. Effect of flow rate in the productivity of continuous fed packed reactor of HB1 in the asymmetric reduction of **1** to ***S*-2.**

Reactor flow (µL/min)	Conversion (%)	Productivity (µM of ***S*-2**/min)
200	26	259
100	55	275
50	100	250
20	100	100
10	100	50

Table S6. Reloading of the heterogeneous biocatalysts with NADH for several cycles.

Load	Relative Cofactor adsorption (%)	Relative Enzyme activity (%)
Load 1	100	100
Load 2	85	99
Load 3	100	82

Cofactor was loaded in 10 mM Tris-HCl buffer at pH 7 on HB1. Then, the solid material was washed with 1 M NaCl to remove the bound cofactor. These two steps were repeated three times. The amount of the immobilized cofactor was quantified by spectrophotometric measurements at 340 nm of the supernatants where the non-bound cofactor remained. The activity of Tt-ADH2 was monitored after the different loading steps.

REFERENCES

[1] C. Mateo, J. M. Palomo, M. Fuentes, L. Betancor, V. Grazu, F. López-Gallego, B. C. C. Pessela, A. Hidalgo, G. Fernández-Lorente, R. Fernández-Lafuente, J. M. Guisán, *Enzyme Microb. Technol.* **2006**, *39*, 274-280.

[2] J. C. S. Dos Santos, N. Rueda, O. Barbosa, J. F. Fernández-Sánchez, A. L. Medina-Castillo, T. Ramón-Márquez, M. C. Arias-Martos, M. C. Millán-Linares, J. Pedroche, M. D. M. Yust, L. R. B. Gonçalves, R. Fernandez-Lafuente, *RSC Advances* **2015**, *5*, 20639-20649.

[3] T. Montes, V. Grazú, I. Manso, B. Galán, F. López-Gallego, R. González, J. A. Hermoso, J. L. García, J. M. Guisán, R. Fernández-Lafuente, *Adv. Synth. Catal.* **2007**, *349*, 459-464.

[4] Y. H. Kim, T. H. Han, H. Cho, S. Y. Min, C. L. Lee, T. W. Lee, *Adv. Funct. Mater.* **2014**, *24*, 3808-3814.

[5] J. Rocha-Martin, D. E. Vega, Z. Cabrera, J. M. Bolivar, R. Fernandez-Lafuente, J. Berenguer, J. M. Guisan, *Process Biochem.* **2009**, *44*, 1004-1012.

[6] E. S. da Silva, V. Gómez-Vallejo, Z. Baz, J. Llop, F. López-Gallego, *Chem. Eur. J.* **2016**, *22*, 13619-13626.

[7] M. M. Bradford, *Anal Biochem* **1976**, *72*, 248-254.

[8] a) J. Rocha-Martín, D. Vega, J. M. Bolivar, A. Hidalgo, J. Berenguer, J. M. Guisán, F. López-Gallego, *Bioresour. Technol.* **2012**, *103*, 343-350;b) J. Rocha-Martín, D. Vega, J. M. Bolivar, C. A. Godoy, A. Hidalgo, J. Berenguer, J. M. Guisán, F. López-Gallego, *BMC Biotechnol.* **2011**, *11*.

[9] J. Rocha-Martín, B. L. Rivas, R. Muñoz, J. M. Guisán, F. López-Gallego, *ChemCatChem* **2012**, *4*, 1279-1288.
